# Increased Incidence of Thyroid Disease in Patients with Celiac Disease: A Systematic Review and Meta-Analysis

**DOI:** 10.1371/journal.pone.0168708

**Published:** 2016-12-28

**Authors:** Xin Sun, Li Lu, Rong Yang, Yanbin Li, Ling Shan, Yang Wang

**Affiliations:** 1 Department of Endocrinology and Metabolism, The People’s Hospital of China Medical University, Shenyang, P.R. China; 2 Department of Endocrinology and Metabolism, The People's Hospital of China Medical University, The People's Hospital of Liaoning Province, No.33 Wenyi Road, Shenhe District, Shenyang 110013, Liaoning Province, P R China; Tulane University, UNITED STATES

## Abstract

The prevalence of thyroid disease is likely increased among individuals with celiac disease (CD). In addition, exposure to gluten-free treatment may be associated with a risk of thyroid disease, but this association remains controversial. A systematic review was performed to evaluate the association between thyroid disease and CD. The articles were obtained from the PubMed, Web of Science, Embase, and Chinese WanFang bibliographical databases for the period up to May 2016. The results were analysed in a meta-analysis with odds ratios (ORs) and corresponding 95% confidence intervals (95% CIs). There were 13 articles in this meta-analysis, including 15629 CD cases and 79342 controls. Overall, the prevalence of thyroid disease in patients with CD was significantly increased compared with that in the control groups (OR 3.08, 95% CI 2.67–3.56, *P*<0.001). Moreover, there was no significant difference in the OR between the gluten-treated and untreated groups (OR 1.08, 95% CI 0.61–1.92, *P* = 0.786). The results of our meta-analysis support the hypothesis that the prevalence of thyroid disease in patients with CD is increased compared with that in controls, which suggests that CD patients should be screened for thyroid disease. The effect of gluten-free treatment on thyroid disease needs further investigation.

## Introduction

Celiac disease (CD) is an autoimmune disorder disease characterized by chronic inflammation and villous atrophy of the small intestine[1]. This disease is caused by the ingestion of gluten-containing foods and other environmental factors. As originally described in the 19th century, CD is characterized by steatorrhoea, vomiting, and cachexia in early childhood. The common clinical manifestations of CD include diarrhoea, emaciation, aphthous stomatitis, and malabsorption, and many patients exhibit only mild or no symptoms at all[2,3]. The working group of the European Society of Paediatric Gastroenterology and Nutrition recommend the use of serological tests as diagnostic criteria for CD. However, the gold diagnostic standard for CD is still the histological changes in the small bowel mucosa. A life-long gluten-free diet is an effective and common method to treat CD[4].

CD has a prevalence of approximately 1% among Western nations[5,6]. The associations of CD with ulcerative colitis, Crohn’s disease, microscopic colitis, autoimmune liver diseases, and several other immune- and non-immune-based diseases in the digestive system are well recognized. However, many diseases outside the digestive system, such as autoimmune thyroid disease, Sjögren’s syndrome, type 1 diabetes mellitus, and Addison’s disease, are associated with CD, in both its overt and silent forms[7]. For example, a meta-analysis based on 26605 patients with type 1 diabetes mellitus revealed a 6.0% prevalence of biopsy-confirmed CD, which was increased compared with the prevalence among healthy controls[8].

Thyroid disease may be related to CD as well. The prevalence of thyroid disease among CD patients was 10.8% in Sweden[9]. Similarly, Hadithi *et al*. found that of 184 patients with CD, 39 (21%) were positive for thyroid antibodies in a Dutch population[10]. Saleem *et al*. also investigated 106 patients with CD in Ireland between 1988 and 2004. The prevalence of thyroid disease was 7%, which was higher than that of other diseases (rheumatoid disease 3%, inflammatory bowel disease 4%, and type 1 diabetes mellitus 2%)[11]. Several studies failed to identify a significant relationship between CD and thyroid disease[12,13]. However, most of these studies were cross-sectional surveys and lacked controls. In addition, exposure to gluten-free treatment may be associated with a risk of thyroid disease[14,15], but this association remains uncertain.

The prevalence of thyroid disease is likely increased among individuals with CD. However, this association remains controversial. The purpose of this study was to evaluate the risk of thyroid disease in patients with CD. A secondary objective was to study the effect of gluten-free treatment on thyroid disease.

## Methods

A completed PRISMA checklist is presented in S1 File.

### Search

A systematic literature search without language restriction was performed independently by two researchers (Y.W. and L.S.) using the PubMed, Web of Science, Embase, and Chinese WanFang bibliographical databases with the words “celiac disease” or “coeliac disease” in combination with the terms “thyroid disease”, or “thyroiditis”, or “thyroid antibody”, or “hypothyroidism”, or “hyperthyroidism” in the title or abstract. The literature was searched for the period up to May 2016. In addition, the reference lists of the retrieved articles were examined to identify additional eligible studies.

### Inclusion criteria

Eligible studies included in this meta-analysis met all the following criteria: (1) all the CD patients were newly diagnosed or untreated with gluten; (2) a case-control or cohort design was used; (3) sufficient data on cases and controls were provided to enable calculation of the odds ratio (OR) with 95% confidence interval (CI) and *P*-value. Reviews, case reports, and letters were excluded from this meta-analysis.

### Data extraction

The following information was extracted from the included studies by two reviewers independently (L.L., Y.B.L.): first author, publication year, region, population, numbers of cases and controls, and details regarding thyroid disease in the cases and controls. Disagreements were resolved by discussion between two reviewers. If a consensus could not be reached, another reviewer settled the disagreement.

The patients in the studies were classified into four groups for further meta-analysis: (1) thyroid disease group, (2) euthyroidism autoimmune thyroid disease group, (3) hypothyroidism group, and (4) hyperthyroidism group. The thyroid disease group was defined as a group of patients with any abnormal thyroid function test, including elevated thyroid antibodies. The euthyroidism autoimmune thyroid disease group was defined as a group of patients with elevated thyroid antibodies in combination with normal thyroid function tests. The hypothyroidism group was defined as a group of patients with hypothyroid function in combination with or without elevated thyroid antibodies. The hypothyroidism group was defined as a group of patients with hyperthyroid function in combination with or without elevated thyroid antibodies.

### Risk of bias in included studies

Two reviewers (L.L., Y.B.L.) independently, but without being blinded to the authors or journals, assessed the risk of bias in the included studies using the Newcastle-Ottawa Scale (NOS)[16]. The NOS is recommended by the Cochrane Handbook for Systematic Reviews of Interventions[17]. Each included study was judged according to 3 domains using the “star system”: representativeness of study group selection (4 items), comparability of groups (two items), and ascertainment of either the exposure or outcome (3 items). NOS scores range from 0 to 9 stars. The studies assessed by both investigators were compared, and disagreement was resolved by consensus.

### Statistical analysis

All data were analysed using STATA software, version 12.0 (Stata Corp LP, TX, USA). The risk of thyroid disease in patients with CD was evaluated by the OR with the corresponding 95% Cl. The significance of the OR was determined using the Z test. Heterogeneity among studies was measured with the *I*^*2*^ index and *P*-value. *I*^*2*^ index values of 25, 50 and 75% were considered to indicate low, moderate and high levels of heterogeneity, respectively. A value of 0% indicated no observed heterogeneity. For low-level heterogeneity, we adopted a fixed-effects meta-analysis. If moderate or high heterogeneity existed, we adopted a random-effects meta-analysis instead of using a fixed-effects model. Next, we examined publication bias using Egger's test and Begg's test. For both tests, *P*<0.1 was considered statistically significant. Furthermore, funnel plots were used to analyse potential publication bias. For other analyses, *P*<0.05 was considered statistically significant.

## Results

### Study inclusion and characteristics

**Fig 1:**
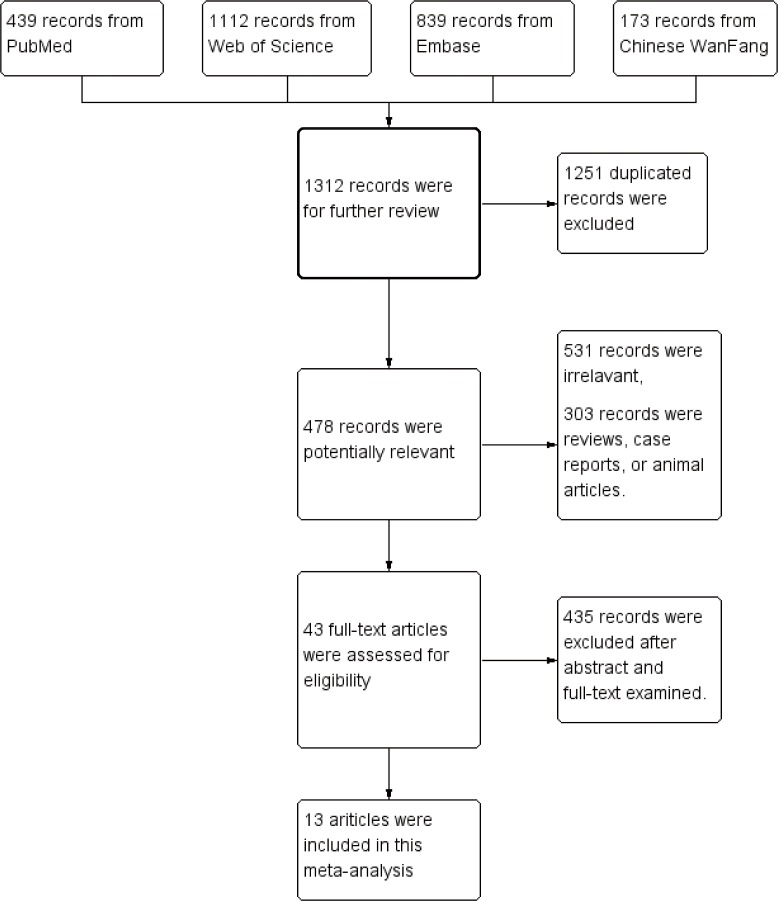
Flow charts showing the detailed procedure for the inclusion or exclusion of studies. Thirteen independent studies were included in this meta-analysis.

Fig 1 summarizes the selection process of studies evaluating the prevalence of thyroid disease in patients with CD. A total of 13 relevant studies with a case-control or cohort design that investigated the relationship between thyroid disease and CD that met the study inclusion criteria were identified.

The initial search retrieved 2563 records from the PubMed, Web of Science, Embase, and Chinese WanFang bibliographical databases, and 478 articles remained after the exclusion of duplicates, reviews, and letters. After screening for eligibility based on the title and abstract, 43 articles were selected. Of these 43 selected articles, 13 articles were included after screening the full text[18–30]. The main reasons for inclusion in the full-text selection are shown in Fig 1. We chose these eligible studies for our meta-analysis, which included 15,629 CD cases and 79,342 controls. The characteristics of the selected studies are summarized in Table 1. Features of the studies comparing the incidence of thyroid disease in CD patients are presented in Table 2.

**Table 1:** Study characteristics of the published studies included in the meta-analysis.

	Region	Design	Population	Case (n)	Control (n)	Outcome
Snook,1989	Oxford	Case-control	Adult	148	300, patients with non-autoimmune gastrointestinal disorders	Hypothyrodism, hyperthyrodism
Sategna-Guidetti, 1998	Torino	Case-control, Prospective cohort	Adult	53 newly diagnosed 132 on a gluten-free diet	170, healthy volunteers, and patients with peptic ulcer, non-ulcer dyspepsia, matched for sex and age	EATD, hypothyrodism, hyperthyrodism
Velluzzi, 1998	Carliari	Case-control	Adult	47	91, healthy subjects, matched for sex, age, and ethnic origin	Thyroid antibodies positive
Kowalska, 2000	Poland	Case-control	Children	34	28, children at similar age with dyspeptic problems	Thyroid antibodies positive
Toscano, 2000	Roma	Case-control	Adolescent	25 newly diagnosed 19 on a gluten-free diet	40, adolescent subjects aged between 14 and 19 years	Thyroid antibodies positive
Ventura, 2000	Trieste, Pisa	Case-control	Mean age 10.1 years	90	90, healthy university students (mean age 20.5 years)	Thyroid antibodies positive
Sategna-Guidetti, 2001	Carliari, Torino, Bologna, Perugia, Napoli	Case-control, Prospective cohort	Adult	241	212, healthy volunteers, and patients with chronic obstructive pulmonary, peptic ulcer, non-ulcer dyspepsia, matched for sex, age, and ethnic origin	EATD, hypothyrodism, hyperthyrodism
Ansaldi, 2003	Torino	Case-control, Prospective cohort	Children	87	199, subjects aged 8 months to 17.3 years, were randomly selected from the areas of patients, and had no immunologic and gastrointestinal disorder.	EATD, hypothyrodism, hyperthyrodism
Guariso, 2007	Padua	Case-control	Children	267	220, healthy children	EATD, hypothyrodism
Elfstrom, 2008	Sweden	Case-control	Children, Adult	14021	68068, matched for age, sex, calendar year, and area of residence	EATD, hypothyrodism, hyperthyrodism
Toumi, 2008	Tunisia	Case-control, Prospective cohort	Adult	56 newly diagnosed 21 on a gluten-free diet	189, normal blood donors (mean age26 years)	Thyroid antibodies positive
Meloni, 2009	Sardinia	Case-control, Prospective cohort	Adult	324	8040, age-matched Sardinian background population	Thyroid antibodies positive
Pals, 2014	Sweden	Case-control, Prospective cohort	12-year-old	242 newly diagnosed 93 on a gluten-free diet	1695, matched for sex, were randomly selected from all cohort members free of celiac disease at the time of diagnosis.	Thyroid antibodies positive

**Table 2:** Details of thyroid disease in patients with celiac disease and controls.

	Thyroid disease in patients with celiac disease (n)	Thyroid disease in control (n)
Snook,1989	Hypothyrodism: 4	Hypothyrodism: 2
	Hyperthyrodism: 2	Hyperthyrodism: 2
Sategna-Guidetti, 1998	EATD: 6	EATD: 8
	Hypothyrodism:3	Hypothyrodism:1
	Hyperthyrodism:1	Hyperthyrodism: 10
Velluzzi, 1998	Thyroid antibody positive: 14	Thyroid antibody positive: 9
Kowalska, 2000	Thyroid antibody positive: 14	Thyroid antibody positive: 2
Toscano, 2000	Thyroid antibody positive: 6	Thyroid antibody positive: 3
Ventura,2000	Thyroid antibody positive: 13	Thyroid antibody positive: 4
Sategna-Guidetti, 2001	EATD: 39	EATD: 8
	Hypothyrodism: 31	Hypothyrodism: 9
	Hyperthyrodism: 3	Hyperthyrodism: 7
Ansaldi, 2003	EATD: 15	EATD: 12
	Hypothyrodism: 9	Hypothyrodism: 7
	Hyperthyrodism: 0	Hyperthyrodism: 0
Guariso, 2007	EATD: 13	EATD: 1
	Hypothyrodism: 13	Hypothyrodism: 0
Elfstrom, 2008	EATD: 17	EATD: 24
	Hypothyrodism: 127	Hypothyrodism: 191
	Hyperthyrodism: 48	Hyperthyrodism: 100
Toumi, 2008	Thyroid antibody positive: 1	Thyroid antibody positive: 3
Meloni, 2009	Thyroid antibody positive: 11	Thyroid antibody positive: 235
Pals, 2014	Thyroid antibody positive: 17	Thyroid antibody positive: 8

Euthyroidism autoimmune thyroid disease: EATD

The Newcastle-Ottawa Scale guidelines are widely used to evaluate case-control and cohort studies for quality. They contain Selection, Comparability, and Exposure categories. Overall, in accordance with the recommended criteria of the Newcastle-Ottawa Scale, the studies included in this meta-analysis were of acceptable quality; therefore, we did not exclude any article from the meta-analysis for quality reasons.

### Meta-analysis results

**Fig 2:**
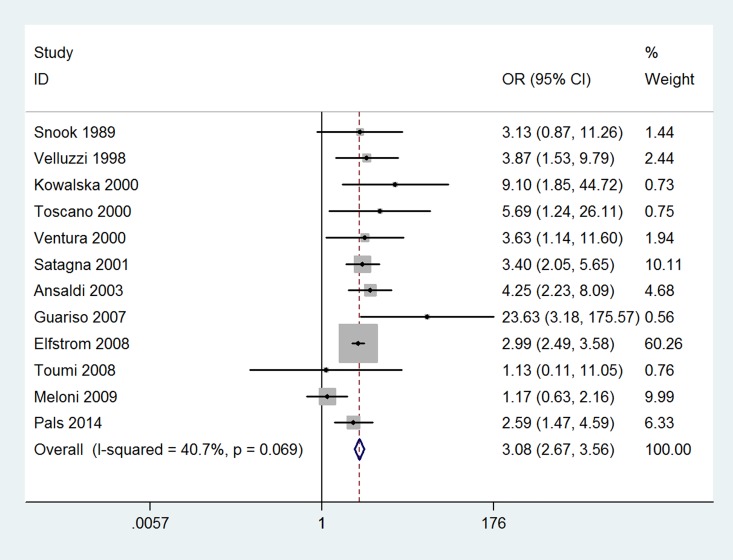
Forest plots for the frequency of thyroid disease in patients with celiac disease compared to that in controls. The diamond represents the pooled OR and 95% CI.

**Fig 3:**
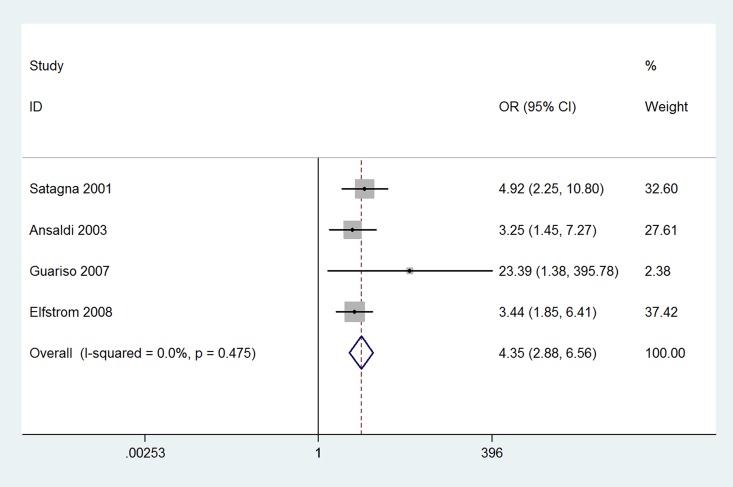
Forest plots for the frequency of euthyroidism autoimmune thyroid disease in patients with celiac disease compared to that in controls. The diamond represents the pooled OR and 95% CI.

**Fig 4:**
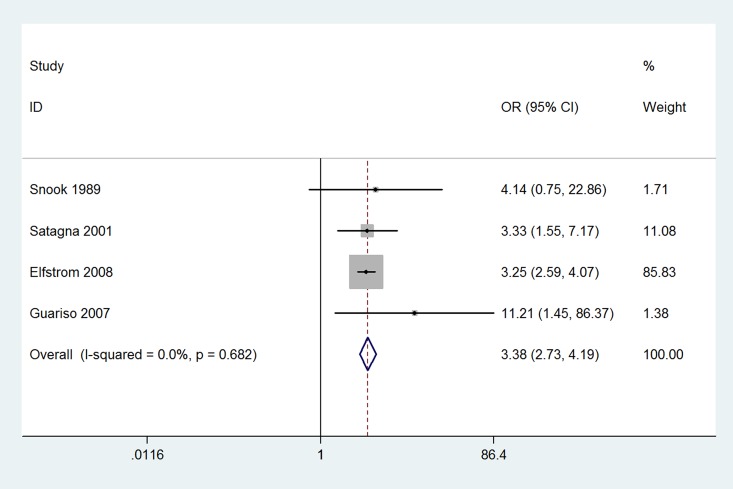
Forest plots for the frequency of hypothyroidism in patients with celiac disease compared to that in controls. The diamond represents the pooled OR and 95% CI.

**Fig 5:**
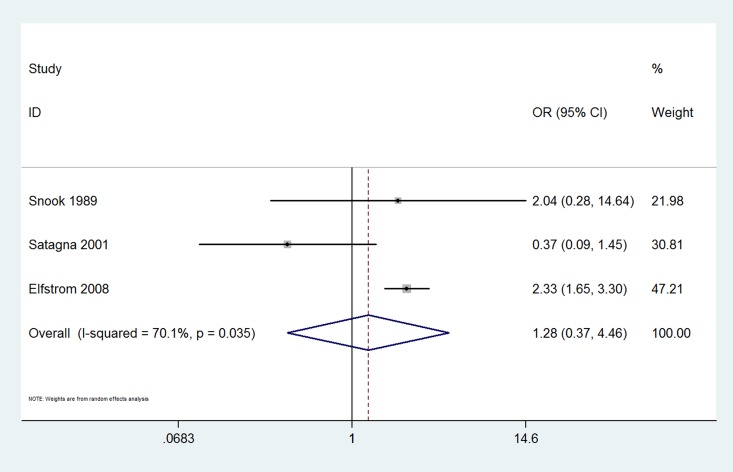
Forest plots for the frequency of hyperthyroidism in patients with celiac disease compared to that in controls. The diamond represents the pooled OR and 95% CI.

**Fig 6:**
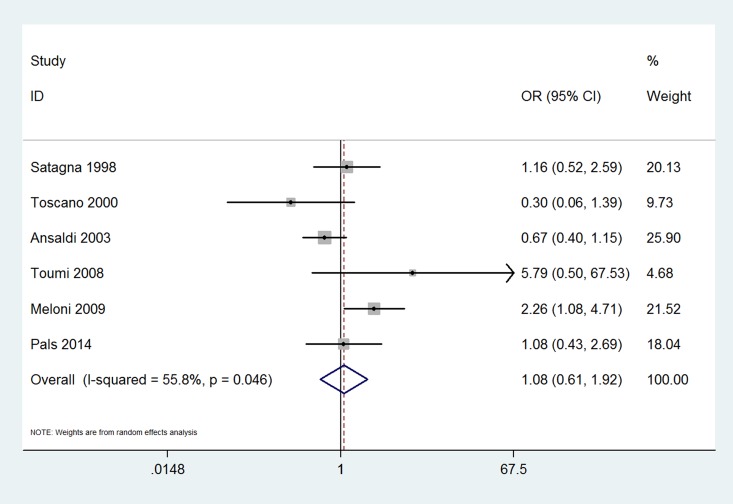
Forest plots for the frequency of thyroid disease in gluten-free-treated patients with celiac disease compared to that in untreated patients. The diamond represents the pooled OR and 95% CI.

The results of this meta-analysis indicated that the prevalence of thyroid disease among CD patients was significantly increased compared with that in the control groups (OR 3.08, 95% CI 2.67–3.56, *P*<0.001). The forest plots for the frequency of thyroid disease in patients with CD compared with controls are presented in Fig 2. The risk of euthyroidism autoimmune thyroid disease in patients with CD was even higher; specifically, it was increased more than four-fold compared with that in patients without CD (OR 4.34, 95% CI 2.88–6.56, *P*<0.001) (Fig 3). The prevalence of hypothyroidism among patients with CD was significantly increased compared with that in the control groups (OR 3.38, 95% CI 2.73–4.20, *P*<0.001) (Fig 4). Unexpectedly, the prevalence of hyperthyroidism in patients with CD was not significantly increased compared with that in the control groups (OR 1.28, 95% CI 0.37–4.46, *P* = 0.693) (Fig 5). There was no heterogeneity in our meta-analysis of the association of euthyroidism autoimmune thyroid disease and hypothyroidism with CD (*I*^*2*^ = 0, *P* = 0.475, 0.682, respectively). Moreover, there was no significant difference in the OR between the gluten-treated and untreated groups (OR 1.08, 95% CI 0.61–1.92, *P* = 0.786) (Fig 6).

### Publication bias

**Fig 7:**
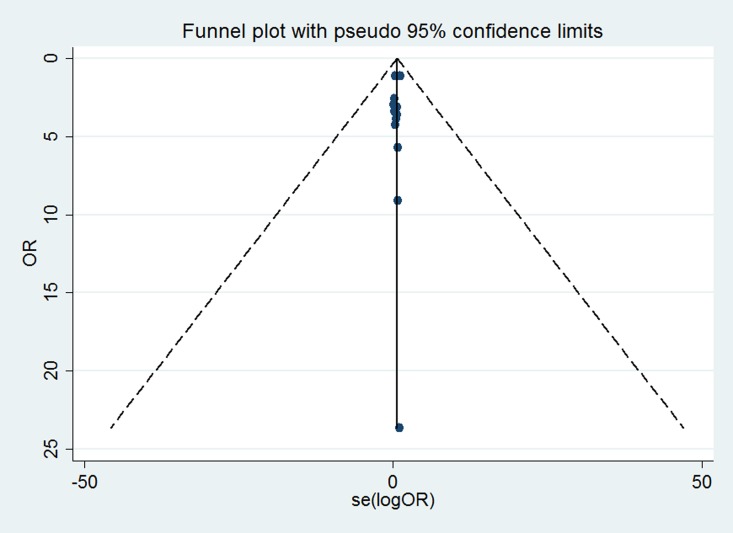
Funnel plot for testing the publication bias of the association between celiac disease and the risk of thyroid disease. Each point represents an individual study on the indicated association. The vertical line indicates the effect size.

We carefully and comprehensively searched the articles in the database. To determine whether potential publication bias existed in the reviewed literature, Begg’s test and Egger’s test were conducted. Begg’s and Egger's tests are used to statistically determine the symmetries of the funnel plots. The results of Begg’s test (*P* = 0.602) and Egger's test (*P* = 0.484) did not suggest the existence of publication bias. The funnel plot is shown in Fig 7.

## Discussion

Some studies have evaluated the risk of thyroid disease in patients with CD, but the results of these studies have not been consistent. Due to the relatively small sample size of these studies, it is difficult to determine the association between CD and thyroid disease. The present systematic review summarized and quantitatively evaluated the evidence for an increased risk of thyroid disease in patients with CD. Thirteen independent, relevant studies with case-control and cohort designs were included in our meta-analysis. The results of this meta-analysis showed that the prevalence of thyroid disease in CD patients was significantly increased compared to that in the control groups (OR 3.08, 95% CI 2.67–3.56, *P*<0.001). The risk of euthyroidism autoimmune thyroid disease among patients with CD was increased more than four-fold compared with that in patients without CD. The prevalence of hypothyroidism in patients with CD was significantly increased compared with that in the control groups. However, there was no significant association between hyperthyroidism and CD. Overall, the heterogeneity in our meta-analysis was low, particularly in that of euthyroidism autoimmune thyroid disease and hypothyroidism (*I*^*2*^ = 0), indicating that the studies included in our meta-analysis were statistically reliable.

Mechanisms that underlie the coexistence of CD and thyroid disease are complex and not yet fully understood. The association can, at least partly, be explained by shared genetic factors. Human leukocyte antigen (HLA) DQ2 and DQ8 haplotypes are over-expressed in CD, and the inheritance of these haplotypes and the associated immunological phenotype may explain this association. HLA-DQ2 and DQ8 also exhibit a weak association with Hashimoto’s thyroiditis; however, the relationship between the HLA-DQ2 haplotype and Graves’ disease is unclear[31–37]. Most hypothyroidism and hyperthyroidism cases are Hashimoto’s disease and Graves’ disease, respectively. In our meta-analysis, there was a significant association between hypothyroidism and CD, but no significant association between hyperthyroidism and CD was observed. Our results are consistent with an earlier mechanistic study.

In addition, the gene encoding cytotoxic T-lymphocyte-associated antigen-4 (CTLA-4) is associated with CD and autoimmune thyroid disease. CTLA-4 is a candidate gene outside the HLA region that is associated with thyroid autoimmunity and exhibits a strong association with CD[38–40]. Moreover, Valentino *et al*. investigated the genotypes of 14 patients with Hashimoto’s thyroiditis compatible with CD. Three patients had DQ heterodimers A1*0501, B1*0201, four had DRB1*04, and one had A1*0101, B1*0501. An increased density of γδ+ T-cell receptor-bearing intra-epithelial lymphocytes was observed in 6 patients. Mucosal T cell activation (presence of interleukin 2 (IL2) receptors (CD25) on lamina propria T cells and/or expression of HLA-DR molecules on crypt epithelial cells, both typical of CD) was noted in those 6 patients. Additionally, in 4 of 6 patients, HLA genotypes associated with CD (three with DRB1*04, DQB1*03 and one with DQA1*0501, DQB1*02) were described[41].

There has been significant debate regarding whether a gluten-free treatment in CD protects against thyroid disease or alters the natural history of the disease. In addition to the studies in this meta-analysis, Cooper *et al*. concluded that gluten-free treatment did not delay the development of thyroid disease and had little ameliorating effect on the disease course, aside from an occasional improvement in atopy[42]. In addition, Viljamaa *et al*. and Mainardi *et al*. both reported no association between duration of gluten exposure in adult CD patients and thyroid disease risk[13,43]. All these related studies lacked a strict design, and randomized controlled trials investigating the effect of gluten on thyroid disease in CD patients were absent. Due to the limited data on the effect of gluten on thyroid disease, only 6 articles were included in our meta-analysis. In our study, we found no significant difference in thyroid disease between the gluten-treated and untreated groups.

## Limitations

The main purpose of this meta-analysis was to investigate the risk of thyroid disease in patients with CD compared with controls using statistical methods. However, there were some limitations in our meta-analysis. First, CD is more common in Western countries than Asian and African countries, and all the studies in our meta-analysis are from Western countries. The relationship between thyroid disease and CD in other ethnic groups and regions was not investigated in this meta-analysis. Second, due to the lack of randomized controlled trials investigating the effect of gluten on thyroid disease in CD patients, the studies included in this meta-analysis were only case-control and retrospective cohort studies. Evidence of an effect of gluten on thyroid disease in this meta-analysis was not strong, and more studies are required. Furthermore, because the studies included were performed at highly diverse time points over approximately 30 years, the variability of the assays used in each study may have influenced the results. Therefore, the results of this meta-analysis should be interpreted with caution.

## Conclusions

In conclusion, the results of our meta-analysis support the hypothesis that the prevalence of thyroid disease, especially euthyroidism autoimmune thyroid disease and hypothyroidism, in patients with CD is increased compared with that in controls, which suggests that CD patients should be screened for thyroid disease. Moreover, the effect of gluten-free treatment on thyroid disease needs further investigation.

## Supporting Information

S1 File PRISMA Checklist. (DOC)

S2 File Search strategy of data in PubMed. (DOC)
