# Supplementary material for: Increased Incidence of Thyroid Disease in Patients with Celiac Disease: A Systematic Review and Meta-Analysis
Source: PLoS One. 2016 Dec 28;11(12):e0168708. doi: 10.1371/journal.pone.0168708 (PMC5193514; doi:10.1371/journal.pone.0168708)
Supplement: S2 File — (DOC) [file pone.0168708.s002.doc]

((celiac disease[tiab]) OR coeliac disease[tiab] ) AND (((((thyroid disease[tiab]) OR thyroiditis[tiab]) OR thyroid antibody[tiab]) OR hypothyroidism[tiab]) OR hyperthyroidism[tiab])
